# Supplementary material for: Resolving the full spectrum of human genome variation using Linked-Reads
Source: Genome Res. 2019 Apr;29(4):635–45. doi: 10.1101/gr.234443.118 (PMC6442396; doi:10.1101/gr.234443.118)
Supplement: Supplemental Material [file supp_gr.234443.118_Supplemental_longranger-2.2.zip › longranger-2.2.tar.bz2!/lr2.2-public/lib/stan/cmdstan/stan/doc/api/html/structstan_1_1gm_1_1sample.html]

Stan: stan::gm::sample Struct Reference


|  |  |
| --- | --- |
|  | Stan  2.5.0  probability, sampling & optimization |


- Main Page
- Related Pages
- Namespaces
- Classes
- Files

- Class List
- Class Index
- Class Hierarchy
- Class Members

All Classes Namespaces Files Functions Variables Typedefs Enumerations Enumerator Friends Macros Pages

- stan
- gm
- sample

Public Member Functions |
Public Attributes |
List of all members

stan::gm::sample Struct Reference

`#include <ast.hpp>`

|  |  |
| --- | --- |
| Public Member Functions | |
|  | sample () |
|  | |
|  | sample (expression &e, distribution &dist) |
|  | |
| bool | is\_ill\_formed () const |
|  | |

|  |  |
| --- | --- |
| Public Attributes | |
| expression | expr\_ |
|  | |
| distribution | dist\_ |
|  | |
| range | truncation\_ |
|  | |

## Detailed Description

Definition at line 818 of file ast.hpp.

## Constructor & Destructor Documentation

|  |  |  |  |  |
| --- | --- | --- | --- | --- |
| stan::gm::sample::sample | ( |  | ) |  |

Definition at line 1361 of file ast\_def.cpp.

|  |  |  |  |
| --- | --- | --- | --- |
| stan::gm::sample::sample | ( | expression & | *e*, |
|  |  | distribution & | *dist* |
|  | ) |  |  |

Definition at line 1363 of file ast\_def.cpp.

## Member Function Documentation

|  |  |  |  |  |
| --- | --- | --- | --- | --- |
| bool stan::gm::sample::is\_ill\_formed | ( |  | ) | const |

Definition at line 1368 of file ast\_def.cpp.

## Member Data Documentation

distribution stan::gm::sample::dist\_

Definition at line 820 of file ast.hpp.

expression stan::gm::sample::expr\_

Definition at line 819 of file ast.hpp.

range stan::gm::sample::truncation\_

Definition at line 821 of file ast.hpp.

---

The documentation for this struct was generated from the following files:

- src/stan/gm/ast.hpp
- src/stan/gm/ast\_def.cpp


---

[ Stan Home Page ]

*© 2011–2014,
Stan Development Team.*
